# Is it worth it? Patient and public views on the impact of their involvement in health research and its assessment: a UK‐based qualitative interview study

**DOI:** 10.1111/hex.12479

**Published:** 2016-06-24

**Authors:** Joanna C. Crocker, Anne‐Marie Boylan, Jennifer Bostock, Louise Locock

**Affiliations:** ^1^ NIHR Oxford Biomedical Research CentreOxfordUK; ^2^ Health Experiences InstituteNuffield Department of Primary Care Health SciencesUniversity of OxfordOxfordUK; ^3^ Health Experiences Research GroupNuffield Department of Primary Care Health SciencesUniversity of OxfordOxfordUK; ^4^ NIHR Collaboration for Leadership in Applied Health Research and Care OxfordOxford Health NHS Foundation TrustOxfordUK; ^5^ UK

**Keywords:** evaluation, impact, patient involvement, public involvement, user involvement

## Abstract

**Background:**

There are mounting calls for robust, critical evaluation of the impact of patient and public involvement (PPI) in health research. However, questions remain about how to assess its impact, and whether it should be assessed at all. The debate has thus far been dominated by professionals.

**Objective:**

To explore the views of PPI contributors involved in health research regarding the impact of PPI on research, whether and how it should be assessed.

**Design:**

Qualitative interview study.

**Setting and participants:**

Thirty‐eight PPI contributors involved in health research across the UK.

**Results:**

Participants felt that PPI has a beneficial impact on health research. They described various impactful roles, which we conceptualize as the ‘expert in lived experience’, the ‘creative outsider’, the ‘free challenger’, the ‘bridger’, the ‘motivator’ and the ‘passive presence’. Participants generally supported assessing the impact of PPI, while acknowledging the challenges and concerns about the appropriateness and feasibility of measurement. They expressed a range of views about what impacts should be assessed, by whom and how. Individual feedback on impact was seen as an important driver of improved impact and motivation to stay involved.

**Conclusions:**

While there appears to be widespread support for PPI impact assessment among PPI contributors, their views on what to assess and how are diverse. PPI contributors should be involved as equal partners in debates and decisions about these issues. Individual feedback on impact may increase PPI contributors’ potential impact and their motivation to stay involved.

## Introduction

Public involvement is defined by NIHR INVOLVE, the National Institute for Health Research patient and public involvement advisory group, as ‘research being carried out with or by members of the public, rather than to, about or for them’. Members of the public include patients, potential patients, carers and people who use health and social care services as well as people from organizations that represent people who use services.^1^ For this reason, it is also often referred to as ‘patient and public involvement’ (PPI). Many different terms are used internationally to describe patients and members of the public involved in research, such as ‘lay representative’, ‘patient partner’ and ‘public adviser’. In this study, we adopt the term ‘PPI contributor’ to avoid implying either that the small number of individuals typically involved in research can represent the diversity of perspectives among patients and the public or that the role of PPI contributors can always be described as a partnership.^2^

The evidence base for the impact of PPI in health research is weak and patchy,^3^, ^4^, ^5^ and there are concerns about its implementation without a thorough justification and understanding of its impact. Recently, there have been calls to improve this evidence base and develop better methods to capture, assess and report the impact of PPI.^6^, ^7^, ^8^, ^9^, ^10^ Frameworks such as the Public Involvement Impact Assessment Framework (PiiAF)^11^ and Guidance for Reporting the Involvement of Patients and the Public (GRIPP)^10^ have been developed, and in‐depth realist evaluations have begun to shed light on what works, for whom, under what circumstances and why.^12^, ^13^ There is a general consensus that although PPI has intrinsic value, it should be scrutinized and evaluated,^14^, ^15^, ^16^, ^17^ although not everyone agrees that its impact should be quantitatively measured.^18^ However, the debate has been dominated by professionals, with little input from patients and members of the public. It has been argued that we need open and honest debate about what is meant by the need to assess the impact of PPI, including who benefits from the assessment and why PPI is being done, before we can conclude that assessment is necessary and determine how to do it.^19^ This paper contributes a diverse range of patient and public views to the ‘impact debate’, as well as shedding light on mechanisms of impact that have so far been underexplored.^8^

As part of a wider study exploring views and experiences of patient and public involvement across the UK,^20^ here we report on PPI contributors’ thoughts and perspectives on the impact of PPI on research and its assessment. We define ‘impact’ as any effect, positive or negative, that PPI contributors have on research processes, outputs and outcomes, including both an individual's impact within a given project, and the impact of PPI more generally on research and research culture. Although not the focus of this study, the impact of PPI on the people involved (PPI contributors and researchers) is another important aspect of impact which merits investigation.^11^ We define the ‘assessment’ of impact as any attempt to judge, either qualitatively or quantitatively, the effect that PPI has on a research project or research more generally. Because an extensive range of impacts of PPI has already been identified and reported,^3^, ^4^, ^12^, ^13^, ^21^ we only briefly describe the impacts identified by participants in this study, focusing more on the mechanisms and assessment of impact.

## Methods

We recruited a maximum variation sample of patients and members of the public from across the UK. Advertisements were sent to universities and clinical research networks and were distributed among the authors’ professional networks and at PPI conferences. The participants had been involved in medical, health or health‐related research for various lengths of time (see Table 1) in a range of different types of research, from qualitative studies to international clinical trials.

**Table 1:** Self‐reported characteristics of interview participants (*N* = 38)

Characteristics	Number of participants
Male	20
Female	18
Age	
18–44 years	5
45–64 years	17
65+ years	16
PPI role^a^	
Patient	24
Carer	9
Dual patient and carer	1
Member of the public	4
Experience of involvement in research	
5 years or less	13
5–10 years	12
More than 10 years	13

a *Participants preferred many different role names, but for the purposes of this paper, we have grouped them into these four categories.

Ethical approval for the study was granted by the Berkshire Research Ethics Committee (ref:12/SC/0495). Five patients and members of the public with PPI experience (including JB) were involved in the study through an advisory group which also included researchers, clinical staff and representatives from PPI organizations. They advised on sampling, recruitment, the interview guide and themes emerging from the analysis. Two of them also participated in the study.

Participants took part in one semi‐structured narrative interview with AMB or LL, the wider findings of which are available on the health website.^20^ Here, we focus specifically on their views about the impact of PPI on research, elicited using a range of prompts (Box 1).

Box 1 **Topics covered by the interview guide**

**Table:** 

Do you feel your involvement has made a difference so far?
What's changed because of your involvement? How/why?
How can we improve the impact of PPI?
Are there any types of research or parts of research where PPI isn't useful?
There's a continuing debate about how we judge the success of PPI in research and how we measure its impact. Do you have any thoughts about that?
Do you think we need to measure PPI or capture its impact?
Do you have any experience of measuring PPI impact?

The interviews were video or audio‐recorded and transcribed verbatim. LL coded the data with NVivo software (QSR International, Melbourne Australia) using a coding framework developed jointly with AMB. Coding was an iterative process; as new codes were added, previous transcripts were re‐coded. JCC then re‐analysed the coding report on ‘value and impact’, using a more refined coding framework developed in discussion with AMB and LL. JB contributed to an initial outline of the paper and successive drafts to refine the analysis and content of the paper.

## Results

### Participants

Thirty‐eight participants consented to be interviewed (see Table 1). Four participants had progressed from a lay PPI role to a professional research or research support role since becoming involved in research.

### Themes

Four broad themes emerged from the analysis and are presented below: (i) the impact(s) of PPI on research; (ii) PPI roles and mechanisms of impact; (iii) the question of whether or not the impact of PPI should be assessed; and (iv) how the impact of PPI should be assessed.

#### What is the impact of PPI on research?

Participants gave many examples of impact or potential impact on research, including shaping initial research questions and ideas, choosing outcome measures that are relevant and meaningful to patients, ensuring the efficient delivery of research, helping to solve ethical dilemmas, improving the way information is communicated to patients, optimizing the recruitment of participants and their experiences of taking part, collecting and analysing research data, and disseminating research findings to patients and the public. Some gave concrete examples of their own impact on such processes:

…it [study] went to ethics and got rejected, and I can remember the PI [Principal Investigator] coming to me on an email saying, ‘Help!’ ….Frankly, the ethics committee was completely right, the patient information sheet was a mess. It didn't take long to actually sort it out…introducing ‘clarity’ is what it's about….And it sailed through ethics the second time round. (P26, patient)

Participants did not frame any of the described impacts on research as negative, including when PPI resulted in research proposals being abandoned:

I said, “I'm thinking about the money you're going to be spending on this … piece of research.” I said, “I think it's a waste of time, I don't think it's going to work the way you want it to pan out”. Anyway they stopped it… (P22, carer)

Importantly, impact did not always mean ‘change’; it could mean validating an existing idea:

I think you're often a sounding board for people who've already got ideas about how a piece of research might run… but they need someone to say, “Yeah absolutely that really, really is important to us.”…. A reassurance that, you know, what you have in mind actually is valid, important… It's not always adding new things; it may be reinforcing what's already there. (P40, patient)

Although convinced that PPI benefited research, some participants found it hard to pin down the impact of their own involvement:

I don't actually know what impact I've had on any of it. No, now you come to talk about it, I have absolutely no idea if anything I have ever done ‐ in the last eight years ‐ has been of any value to anyone at all, which is actually quite a sobering thought. (P32, patient)

Some participants encouraged other PPI contributors to seek feedback from researchers to increase the value of their contributions. Impact could change over time and be enhanced through ongoing, reciprocal feedback:

As you're leaving the meeting say to the Chair, “Were those contributions helpful to the meeting?” and get them to tell you if they were. And say to him, “Look I'm new to this, give me advice.” I found sometimes that some professionals have said to me, “We'd love it if you asked questions around such and such.” (P27, patient)

#### Roles and mechanisms of impact

Participants described themselves and their peers as fulfilling a variety of roles in terms of their ‘added value’. We grouped these into six broad categories: the expert in lived experience, the creative outsider, the free challenger, the bridger, the motivator and the passive presence (Table 2). These are fluid and not mutually exclusive, for example the bridger may connect researchers to the patients they aim to benefit, thus potentially increasing their motivation.

**Table 2:** PPI contributors’ perceived roles and mechanisms of impact

Perceived role	Proposed mechanism of impact	Illustrative quote(s)
The expert in lived experience	Through their lived experience of a condition, PPI contributors are able to consider the acceptability and feasibility of research proposals for the target population	‘And many of these researchers and scientists only ever see [motor neurone disease] down a microscope, put in a Petri dish. But its meaning and its effect is unknown to them. I, on the other hand, am an expert of what it is to live and to die with motor neurone disease. And that does have a value to research’. (P03, carer) ‘…there was a piece of research about eating in dementia…and they were thinking, is it better for a dementia person to eat at lunch time rather than in the evening? […] And I said… “I don't think this study's going to work, this food study… because a dementia person will not sit down at meal times, at lunchtime, and eat a full meal.” […] And they must have listened because like I said they did take it off, they didn't bother with it’. (P22, carer)
The creative outsider	PPI contributors bring a fresh perspective from outside the research system, and can help to solve problems by thinking ‘outside the box’	‘By taking non‐experts into any field you can possibly get a whole leap forward because somebody suggests you look outside the box and you look at it from a different perspective’. (P11, patient/carer) ‘Members of the public – because of their different understandings – can come out with the most bizarre suggestions. But also, the most incredible suggestions that actually are the most important’. (P32, patient)
The free challenger	PPI contributors are able to challenge researchers without fear of consequences	‘We can ask the elephant in the room question. We can say, “Well why not? Why can't you do this? Well why can't you do it that way?” We're not employed, we don't have to worry about the hierarchy in our jobs…We can challenge from a purely interested point of view, not worrying about the bosses or the NHS or anything really’. (P31, patient) ‘A lot of academics in that group would have a stake in going forward with the leader because their jobs… it depends on being seen in a good light by the leader. The great advantage of the citizen researcher is that we don't. We are volunteers, we can speak truth to authority without danger of retribution…’ (P24, public)
The bridger	PPI contributors bridge the communication gap between researchers and patients or the public, making research more relevant and accessible	‘That's one of the main contributions that lay people can make, “What does that mean? What does that mean for me? What does that mean for my friends? What will it mean for the future? Will it make me better? Will it make my auntie better?” […] And sometimes clinical researchers may not… have thought of the issues with that simplicity so I'm I suppose making a case for public and patient involvement to make research as simple as possible in how to understand it, what it's going to achieve and how you tell the public about it’. (P18, public)
The motivator	PPI contributors increase researchers’ motivation/enthusiasm, for example by emphasizing how the research will benefit people.	‘… I've seen researchers get really very excited about how real the whole thing seems as opposed to sort of theoretical and academic. So they can start to see how the research they're doing is really going to benefit people so it [PPI] gives a sort of extra sort of brilliance to it, it makes it more exciting and engaging’. (P12, patient)
The passive presence	PPI contributors can change the way that professionals think just by being present at meetings.	‘Sometimes, even if we're just there as a listener, not as an active contributor, but the professionals know that we are there, and they try to think from our perspective as well’. (P06, carer) ‘…Afterwards someone says, “You have no idea the difference that your being there, just being in a room, has made… People are stopping and listening to each other, not just you, in a different way.”’(P40, patient)

We see these roles as functioning within a team of research professionals and PPI contributors, as participants stressed the importance of teamwork in driving impact:

It's a different perspective. It is not better. It is not inferior. We need the doctor. We need the scientist. We need the neurologist. We need the PPI. We need a team. And it's a team effort that will eventually yield the results. (P03, carer)

#### Should the impact of PPI be assessed?

Participants were generally in favour of assessing the impact of PPI, to improve the way it is done, to convince sceptical researchers of its benefits or to reduce tokenistic PPI, to justify the cost of PPI and to increase funding for PPI:

…this is a big investment that we are making, and so we ought to be contributing something. It's not just about having quite a nice time. (P02, carer)

…you need to measure things because of the old adage that what gets measured gets done. And if you don't measure things in some way or other then you have no idea whether you're doing well or doing badly. (P09, carer)

And that's the other thing about impact, you need to demonstrate it, not just to funders, you need to demonstrate it to other patients so they will get involved and think, ‘Yeah I could do this.’ (P40, patient)

Some participants emphasized the need for individual feedback regarding their own impact, as this would improve their contributions and increase their motivation to stay involved:

…people tend to give me an idea of how they're going to use my information, which of course is important because that informs how I react to the next set of reviewing that I do. (P12, patient)

[Feedback from researchers] is a very, very important part of the process that isn't happening. And I think, actually, that may well be one of the major parts of the process that keeps people participating… (P32, patient)

However, there was a widespread acknowledgement that assessing the impact of PPI is challenging, particularly when PPI is a genuinely collaborative venture. Participant 19 (a patient), for example, described themselves as ‘just a little cog’ contributing to ‘moving in the right direction’. Participant 33 (a patient) felt it was ‘more difficult to see a direct result of what you've done the more sophisticated you get with your PPI activity’.

None of the participants appeared to be unequivocally opposed to assessing the impact of PPI. However, some were concerned about assessing impact too simplistically:

…if, for instance, you're holding workshops where people are talking with each other, including researchers and, and service users, it's quite difficult to then pull apart whose contribution made which difference. So those things make it quite complicated. So I think simplistic tools for measuring impact can be quite damaging, because they're not likely to notice it. (P02, carer)

Participant 41 (a patient) also questioned the singling out of PPI members rather than others on the research team: ‘What about the other people whose expertise you're asking? Are you going to measure the impact the statistician had when you asked him or her to help…?’

#### How should the impact of PPI be assessed?

Participants suggested several ‘impacts’ which could be quantitatively measured: success in gaining research funding, research ethics committee approval and participant recruitment rates. One participant proposed that increased demand for PPI in research was itself evidence of its positive impact:

…the biggest indication of our value is that we can't keep up with requests for input. And it's from people that are really good researchers, very well‐known, but also from some of the young researchers who've heard about us and actually have come usually via our website and asked for input… So the proof of the pudding's in the eating and the number of people that…want to buy it. (P31, patient)

However, participants varied in their attitudes towards quantitative measurement of impact. Randomized controlled trials were viewed as the most convincing type of evidence, but not necessarily appropriate for assessing PPI impact:

So I think some of the difficulty of that sort of question is, well, what do we mean by evidence? What do we mean by impact? Who is it we're trying to convince by this evidence? Because actually… if you're trying to convince somebody who only believes in randomised controlled trials then actually we're never going to probably get evidence. Whereas if you're going to get evidence for people who… will consider a broader range of research methods and their findings, then I think we can provide more evidence. (P16, patient)

Qualitative methods were seen as valuable or essential by some participants because of the need to capture unintended as well as intended impacts:

I think [narrative case studies] have got to be part of it and I think they can start to uncover what impact is intended and what impact is not intended as well, which is, I think, something that's quite important in these very complex relationships. (P02, carer)

Some participants spoke of the need to move beyond ‘anecdotal’ evidence of impact, although not everyone agreed that ‘anecdotal’ evidence was a bad thing:

…There's currently a bid out to measure the impact of PPI […] to find tools that measure the impact… Because the only way to get it more embedded is to actually be able to point to something that shows – because at the moment it's dismissed as anecdotal evidence and I don't know why anecdotal evidence doesn't count. (P36, patient)

Although relatively tangible impacts were suggested for assessment, many participants proposed that the ultimate aim of PPI was to benefit patients through improved research:

It's no good just going down some wonderfully enthusiastic path as a researcher which may or may not have an impact on the real world. Far better to say, well I would find it more satisfying to be able to say at the end of it, “This research had an impact on hospital practice or what GPs do.” To me that is such a valuable output from research that it's well worth taking a little time at the start to get lay input. (P39, patient)

There was some discussion about who should be involved in assessing impact. Some PPI contributors suggested they could keep a record of their own impact, and some said it was important to ask researchers:

You're going to have to ask investigators, particularly chief investigators, principal investigators, the ones who actually put studies together in detail, what their perception of the value is and hopefully they will do more than give the nominally appropriate answer. (P26, patient)

## Discussion

### Main findings

Participants in this study overwhelmingly expressed the view that PPI has or should have a beneficial impact on health research, describing various positive impacts and potential impacts on research processes which mirror those identified in systematic reviews.^3^, ^4^ This is consistent with the finding of a recent UK consensus study that the majority of public participants felt that PPI leads to research of greater quality and relevance – a view only shared by a minority of academics.^16^ In keeping with a qualitative study of PPI in clinical trials,^2^ none of the participants reported negative impacts of PPI on research, although some expressed uncertainty about the impact of their own involvement. In our study, this seemed to be in part because of a lack of individual feedback on impact within specific research projects. Such individual feedback was seen as an important driver of impact improvement and motivation to stay involved in research.

Participants described various impactful roles, which we have referred to as the ‘expert in lived experience’, the ‘creative outsider’, the ‘free challenger’, the ‘bridger’, the ‘motivator’ and the ‘passive presence’. In practice, these roles may frequently overlap and PPI contributors may embody all of them at different times throughout the life of a research project. We hope that this suggested typology helps towards better understanding some of the mechanisms leading to PPI impact, and towards clarifying what types of impact PPI contributors and researchers want PPI to have in research – a crucial step in determining what impacts to assess and how.^15^ Determining which of these roles will be prioritized at the outset of research projects could help research teams recruit PPI contributors with the experience, attributes or skills required to fulfil these roles, and could help to clarify goals at an early stage. This may in turn help to increase the perceived value and impact of PPI, as there appears to be a link between chief investigators having goals for PPI in clinical trials and believing that PPI made a positive difference.^2^

Participants generally supported the idea of PPI impact assessment, although some questioned whether or not it was possible to do well, given the complex nature of PPI. It was pointed out that the more PPI resembles a partnership, with PPI contributors being part of a team alongside researchers and other professionals, the more difficult it is to isolate the impact of the PPI. This is an important consideration given the indication that a ‘fully intertwined’ partnership approach leads to greater positive impact.^13^ There was some concern about the dangers of oversimplifying the assessment of PPI impact and producing distorted results. These views reflect those of many academic and non‐academic stakeholders who took part in a consensus study about PPI evaluation: 89% expressed the view that PPI impact assessment was very or fairly important, although many acknowledged that such assessment was methodologically challenging.^16^

The participants in our study expressed a range of different views about what impacts should be assessed, by whom and how. Given that many of the people we interviewed were involved in quantitative clinical studies, it is perhaps not surprising that there was general acceptance of a biomedical hierarchy of evidence (with randomized controlled trials at the top) and acceptance of a discourse about the need to demonstrate effectiveness in fairly narrow utilitarian terms. Yet people we interviewed also expressed reservations about applying this paradigm to PPI – a complex social process – and its possible unintended consequences.

## Strengths and limitations

To our knowledge, this is the first in‐depth, UK‐wide exploration of PPI contributors’ views on PPI impact assessment in health research. We hope the findings will be considered alongside the predominantly professional views in current literature. The purposive sampling strategy and one‐to‐one interviews yielded rich data on a diverse range of views and experiences. In addition, the authors come from different paradigmatic stances and hold differing views on whether and how PPI impact should be assessed. The third author is a PPI contributor herself and was involved in designing the study and interpreting data.

Our study has several limitations. First, the interviews required participants to recall past experiences of PPI, which for some participants stretched back over many years. It may be that in some cases a lack of information, such as examples of participants’ own impact or examples of negative impact on research, reflects recall difficulties rather than evidence of absence. Second, participants may have felt reluctant to speak critically about an enterprise they have bought into. The interviewers themselves were researchers (albeit non‐clinical), and this may have influenced the way participants spoke about PPI impact and its assessment, for example, softening or omitting disagreement with prevailing academic views (such as the discourse of evidence‐based practice and the hierarchy of types of evidence). There remains ample evidence that PPI often takes place in a context of unequal power relations. As Gibson, Lewando‐Hundt and Blaxter argue, in some circumstances PPI ‘offers relatively limited opportunities to influence decision making or alter agendas’, and can take the form of a ‘weak public, lacking in general participatory parity and therefore unable to challenge the boundaries and discourse [of the boards]’.^22^ Although their study focused on PPI in service networks rather than research, their conclusion is strongly resonant.

Finally, the interview guide covered many different topics, of which PPI impact was only one and probing on this specific topic was therefore limited. In retrospect, specific probing about the potential negative impacts of PPI may have yielded useful findings to complement the positive accounts of PPI impact elicited.

## Implications

We believe that there are several important implications of our findings. First, PPI contributors should be involved as equal partners in debates and decisions about what impacts to assess, why and how. Just as researchers hold a range of differing views about PPI impact assessment,^16^ so too do PPI contributors, and their representation in such debates and decisions should reflect this diversity. Second, the six PPI roles we conceptualized may aid research teams in planning PPI, recruiting and working with PPI contributors. Prospective, in‐depth research such as ethnography may help to further uncover the mechanisms by which these roles lead to impact on research. Third, while the assessment of some types of PPI impact may be methodologically challenging, documenting the contributions of individual PPI contributors and the incorporation of these contributions into research projects may be relatively feasible, and would be welcomed by many PPI contributors who wish to see what difference they are making and increase their potential impact. Assessing the impact of PPI in isolation may be perceived as discriminatory,^16^ therefore, we would encourage researchers to discuss with their PPI contributors whether such an approach would be helpful, and if so, how it should be done.

## Conflict of interests

The authors declare no conflict of interests.

## Source of funding

This research was funded by the National Institute for Health Research (NIHR) Oxford Biomedical Research Centre based at Oxford University Hospitals NHS Trust and University of Oxford. JC and LL are supported by NIHR Oxford Biomedical Research Centre Fellowships. AMB is supported by the NIHR Collaboration for Leadership in Applied Health Research and Care Oxford at Oxford Health NHS Foundation Trust. The views expressed are those of the authors and not necessarily those of the NHS, the NIHR or the Department of Health.
